# Imatinib mesylate in combination with pembrolizumab in patients with advanced *KIT*-mutant melanoma following progression on standard therapy: A phase I/II trial and study protocol

**DOI:** 10.1097/MD.0000000000027832

**Published:** 2021-12-10

**Authors:** Ikuko Hirai, Keiji Tanese, Keitaro Fukuda, Takayuki Fusumae, Yoshio Nakamura, Yasunori Sato, Masayuki Amagai, Takeru Funakoshi

**Affiliations:** a Department of Dermatology, Keio University School of Medicine, Tokyo, Japan; b Department of Preventive Medicine and Public Health, Keio University School of Medicine, Tokyo, Japan.

**Keywords:** anti-PD-1 antibody, imatinib mesylate, KIT, metastatic malignant melanoma, pembrolizumab

## Abstract

**Introduction:**

Genetic alterations of *KIT* gene are known to be one of the major causes of melanoma. Those are more common in the mucous and acral subtypes and KIT is regarded as major oncogene in Asian melanomas, where the prevalence of these subtypes is high. Up to date, several clinical trials have been conducted to target *KIT* gene alterations in melanoma with unsatisfied efficacies. Imatinib mesylate, a small-molecule inhibitor of the KIT tyrosine kinase, provides a rapid but not durable clinical response in *KIT*-mutant melanoma. Meanwhile, recent basic and clinical evidence have revealed another aspect of KIT-targeted therapy, namely the enhancement of antitumor activity of immune checkpoint inhibitors. Herein, we designed clinical trial of co-administrating imatinib mesylate and pembrolizumab (anti-PD-1 antibody) to evaluate its safety and efficacy.

**Methods and analysis:**

This is an open-label, single-arm, phase I/II clinical trial involving Japanese patients with metastatic *KIT*-mutant melanoma that are refractory to standard therapy including anti-PD-1 therapy. Phase I study is a dose-escalation study comprising two dose levels of imatinib mesylate (200 and 400 mg/day, respectively) with fixed dose of pembrolizumab (200 mg every 3 weeks) to evaluate safety and tolerability and determine recommended phase II dose. The primary endpoint of the phase II study is the objective response rate after 4 cycles (3 weeks/cycle) of pembrolizumab and imatinib mesylate at the dose determined in phase I, based on RECIST version 1.1. A Simon's minimax two-stage design is employed to test the null hypothesis of a 5% response rate vs 30% alternative, which will be rejected when a lower confidence limit of two-sided 90% confidence interval of true response rate is over than threshold response rate. The secondary endpoints include progression free survival, overall survival, best overall response and incidence of adverse events. Totally, a target size of 22 patients will be expected.

**Discussion:**

If this study shows efficacy and acceptable safety profile, it will contribute to the development of novel treatment option for patients with *KIT*-mutant melanoma that are refractory to standard therapy.

**Trial registration:**

NCT04546074. Date of Registration: September 11, 2020 (https://clinicaltrials.gov/ct2/show/NCT04546074). Date of First Participant Enrollment: December 23, 2020.

## 1 Introduction

KIT is a type III transmembrane receptor. Upon the biding with its ligand, it triggers activation of downstream signalling pathways to promote cell growth, survival, and migration. Gain-of-function mutations of *KIT* gene have been found in several cancers, hence it is recognized as an oncogene. Prevalence of the alteration of *KIT* gene in melanoma is associated with clinical subtypes, which is more common in acral and mucosal subtypes.^[1]^ Gene amplification is found in about 26% of mucosal and 27% of acral types, and mutation is found in about 25% of mucosal and 21% of acral types.^[2]^ As acral and mucosal type melanomas account for the majority in Asian melanoma, *KIT* gene is regarded as major oncogene causing melanoma in Asia.^[2–4]^

KIT-targeted therapies have not shown satisfactory effect in melanoma to date. While several KIT tyrosine kinase inhibitors (KITi) have shown certain tumor shrinkage effect on *KIT*-mutant melanoma as a monotherapy (ORR; 16–29%), those effects were non-durable (median PFS; 2.8–3.7 months).^[5–11]^ Meanwhile, several preclinical data have shown the other role of KITi that they do not only target tumoral *KIT* gene products but also modulates key signalling pathways of immune cells that are involved in cancer immunosurveillance.^[12]^ In addition, KITi may promote antitumor immunity and exhaust synergistic anti-tumor effect in combination with immune checkpoint inhibitors (ICIs).^[13,14]^ Furthermore, a melanoma patient with *KIT* mutation who was refractory to anti-PD-1 monotherapy has been successfully treated with a combined administration of imatinib mesylate and anti-PD-1 antibody.^[15,16]^

Based on these promising evidences of ICI and KITi combination, we proposed to conduct the phase I/II clinical trial of imatinib mesylate (KITi) with pembrolizumab (anti-PD-1 antibody) for *KIT*-mutant melanoma patients who progressed after ICIs.

## 2 Methods/design

### 2.1 Objectives and study setting

This study is a single-arm, open-label, phase I/II clinical trial aiming to evaluate the safety, tolerability, and response rate data of imatinib mesylate and pembrolizumab in patients with refractory *KIT* mutant malignant melanoma. The recruitment of study participants started in September 2020 and will continue until a total of 22 participants are registered.

### 2.2 Study design

The phase I study is a dose-escalation study comprising two dose levels of imatinib mesylate (200 and 400 mg/day, respectively) with fixed dose of pembrolizumab (200 mg every 3 weeks) to evaluate safety and tolerability and identify maximum tolerated dose/administered, and determine recommended phase II dose (RD).

In phase II study, a Simon's minimax two-stage design is employed to test the null hypothesis of a 5% RR vs 30% alternative (1-sided α 0.05, power 80%, ≥1/7 RR to continue to total of 3 ≥ 14). The treatment period with the combination therapy of both studies will continue every 21 days for up to 33 cycles (∼2 years) as long as patients are receiving benefit from treatment and have not had disease progression or met any criteria for study withdrawal.

**Figure 1:**
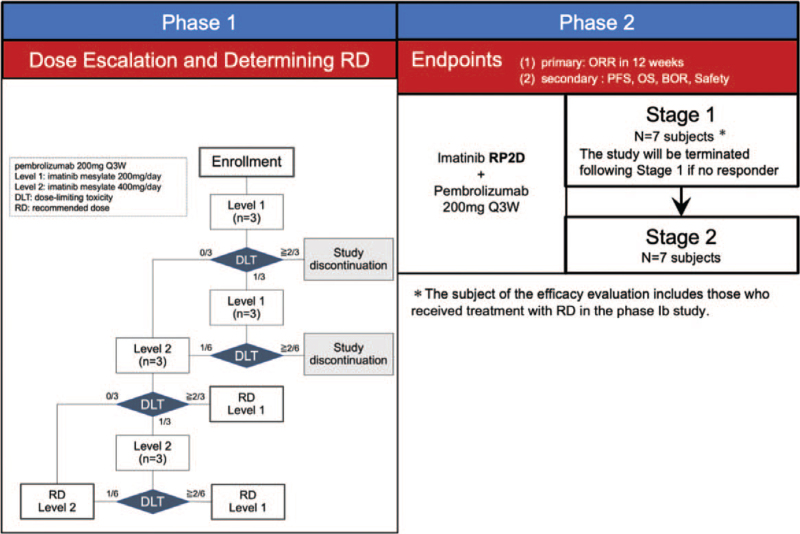
Flow chart of phase I study.

The trial design is depicted in Figure 1.

### 2.3 Inclusion criteria

1. Age 20 years or older on day of signing consent.
2. Histologically confirmed melanoma.
3. Unresectable or metastatic melanoma with *KIT* mutations detected by next-generation sequencing which was performed at clinical laboratories accredited by international standards such as Clinical Laboratory Improvement Amendments (CLIA) and College of American Pathologists (CAP) certification.
4. Progression on treatment with an anti-PD-1/L1 Ab administered either as monotherapy, or in combination with other checkpoint inhibitors or other therapies. PD-1 treatment progression is defined by meeting all the following criteria:
  a. The patient received at least 2 doses of an approved anti-PD-1/L1 Ab.
  b. The patient has demonstrated disease progression after PD-1/L1 Ab as defined by RECIST 1.1.
    The initial evidence of disease progression is to be confirmed by second assessment no less than 4 weeks from the date of the first documented progressive disease, in the absence of rapid clinical progression. This determination is made by the investigator. Once progressive disease is confirmed, the initial date of progressive disease documentation will be considered the date of disease progression.
  c. Progressive disease has been documented within 12 weeks from the last dose of anti-PD-1/L1 Ab or during the treatment. In adjuvant therapy, progressive disease has been documented within 6 months from the last dose of anti-PD-1/L1 Ab or during the treatment.
5. No prior treatment with KIT inhibitors.
6. Completed previous therapies 21 days prior to enrollment on study.
7. The presence of at least one measurable lesion by CT or MRI per RECIST 1.1 criteria as determined by the local site investigator/radiology assessment within 14 days prior to enrollment on study.
8. An Eastern Cooperative Oncology Group (ECOG) performance status 0 to 1.
9. Life expectancy of >90 days
10. No active central nervous system (CNS) metastases. Patients with previously treated brain metastases may participate provided they are stable (without evidence of progression by imaging for at least 28 days before enrollment on study and any neurologic symptoms).
11. If female of childbearing potential, must be willing to use an adequate method of contraception and agree not to breastfeed for the course of the study through 120 days after the last dose of study medication. If male of childbearing potential, must agree to use an adequate method of contraception for the course of the study through 120 days after the last dose of study medication.
12. Laboratory parameters within Protocol-defined range. The screening laboratory tests below must be ≤14 days before enrollment on study.
  a. White blood cell count ≥2000/mm^3^ and absolute neutrophil count ≥1500/mm^3^.
  b. Platelets ≥ 100,000/mm^3^.
  c. Hemoglobin ≥ 9 g/dL.
  d. Aspartate aminotransferase (AST), alanine aminotransferase (ALT) ≤ 150 IU/L.
  e. Bilirubin ≤ 2.0 mg/dL.
  f. Serum creatinine ≤ 1.5 mg/dL.
13. Be willing and able to review, understand and provide written consent before starting therapy.

### 2.4 Exclusion criteria

1. Seropositive for hepatitis B antigen, or hepatitis C antibody.
  Even if negative for HBs antigen, patients who are positive for HBs antibody or HBc antibody and amount of HBV-DNA exceed detection sensitivity are excluded from the study.
2. History of human immunodeficiency virus (HIV) (HIV 1/2 antibodies).
3. History of (no-infectious) pneumonitis that required steroids or present pneumonitis.
4. Ongoing or active infections, symptomatic heart failure, unstable angina pectoris, cardiac arrhythmias, interstitial pneumonia, pneumonitis, or psychiatric disorders that would interfere with cooperation with the requirements of the study.
5. Superior vena cava syndrome, pericardial effusion, pleural effusion, or ascites of grade 3 or higher.
6. Gastrointestinal condition that may affect drug absorption.
7. Receiving or necessary to continue administering CYP3A4 inhibitors or drugs metabolized by CYP3A4.
8. Additional malignancy that is progressing or requires active treatment. Exceptions include cancers that have not recurred for at least 3 years before enrollment on study and early stage cancers (carcinoma in situ or stage 1) treated with curative intent, basal cell carcinoma of the skin, or superficial bladder cancer that has undergone potentially curative therapy.
9. Active autoimmune disease that has required systemic treatment in past 2 years.
10. History of organ transplantation including hematopoietic stem cell transplantation.
11. Receiving chronic systemic steroid therapy. Replacement therapy (e.g., thyroxine, insulin, or physiologic corticosteroid replacement therapy for adrenal or pituitary insufficiency, etc) is not considered a form of systemic treatment.
12. Pregnant women and breastfeeding woman.
13. Having received live vaccine within 30 days before the first dose of study treatment. Examples of live vaccines include, but are not limited to, the following: measles, mumps, rubella, chicken pox, yellow fever, rabies, Bacillus Calmette-Guerin (BCG), and typhoid vaccine. Seasonal influenza vaccines for injection are generally killed virus vaccines and are allowed; however, intranasal influenza vaccines (e.g., FluMist) are live attenuated vaccines and are not allowed.
14. History or current evidence of any condition, therapy, or laboratory abnormality that might confound the results of the study, interfere with the patient's participation for the full duration of the study, or is not in the best interest of the patient to participate, in the opinion of the treating investigator.

### 2.5 Ethics approval and consent to participate

This study is being conducted in accordance with the principles expressed in the Declaration of Helsinki, Japanese Clinical Research Act and SPIRIT (Standard Protocol Items: Recommendations for Interventional Trials) guidelines, and conducted as “Advanced medicine” authorized by the Ministry of Health, Labor, and Welfare (MHLW). The protocol version 1.3 was approved by Keio Certified Review Board (CRB3180017) in November 2019 and MHLW.

### 2.6 Endpoints

The primary outcome of phase I study is the dose limiting toxicities (DLT) rates of pembrolizumab and imatinib mesylate combination therapy. The primary outcome of phase II study is the objective response rate (ORR) of treatment with recommended dose of imatinib mesylate with fixed dose of pembrolizumab in 12 weeks according to Response Evaluation Criteria in Solid Tumors (RECIST) version 1.1. The patient of the efficacy evaluation includes those who received treatment with RD in the phase I study. Secondary outcome measures include progression-free survival (PFS), overall survival (OS), best overall response (BOR), and incidence of adverse events (AEs) graded using the National Cancer Institute (NCI) Common Terminology Criteria for Adverse Events (CTCAE) version 5.0. PFS is defined as the time from date of registration until the earliest date of disease progression or death from any cause, whichever comes first. OS is defined as the time from date of registration to date of death due to any cause. BOR is defined from the overall efficacy determined by the end of this trial according to RECIST 1.1. Determining CR and PR requires radiologic confirmation by continuous evaluation over an interval of 4 weeks.

### 2.7 Treatment in phase I study

Phase I study is 3+3 dose-escalation study comprising two dose levels of imatinib mesylate with fixed dose of pembrolizumab. This study will be broken into 2 levels of 3 to 6 patients each level. The cycle length of regimen is 21 days. Each patient will receive 2 cycles of imatinib mesylate and pembrolizumab combination therapy and assessed for DLT at day 42 (±2). The first level (Level 1) of patients will receive the lowest dosing regimen, oral imatinib mesylate 200 mg quaque die (QD) for 21days and intravenous pembrolizumab 200 mg on day1 every 21 days, with subsequent cohorts receiving increasing doses of imatinib mesylate, 400 mg QD. After the RD has been determined, this dosage scheme will be used to treat patients in phase II study. If patients in phase I show no DLTs followed by non-progressive disease, they will continue with combination therapy for up to 2 years.

Escalation scheme is as follows (Fig. 1):

1. If DLT is not observed in all 3 cases in Level 1, proceed to enrollment of Level 2.
2. If DLT is observed in one of 3 cases in Level 1, add further 3 cases, and
  a. If DLT is observed in 1 of total 6 cases, proceed to enrollment of Level 2.
  b. If DLT is observed 2 or more of 6 cases, the study is discontinued.
3. If DLT is observed 2 of 3 cases in Level 1, the study is discontinued.
  Regarding the transition to Level 2, the evaluation of the investigator will be discussed and judged by Efficacy and Safety Evaluation Committee.
4. If DLT is not observed in all 3 cases in Level 2, dose of Level 2 is determined as RD.
5. If DLT is observed in one of 3 cases in Level 2 cohort, add further 3 cases, and
  a. If DLT is observed in 1 of total 6 cases, dose of Level 2 is determined as RD.
  b. If DLT is observed 2 or more of 6 cases, dose of Level 1 is determined as RD.
6. If DLT is observed 2 of 3 cases in Level 2, dose of Level 1 is determined as RD.
  The evaluation of the RD by the investigator will be discussed and judged by Efficacy and Safety Evaluation Committee.

### 2.8 Definition of DLT

DLT will be assessed by physical examination, observing and questioning patients regarding adverse experiences, and monitoring clinical chemistry and hematology by the treating investigator. DLT is defined as any:

- Any of the Grade 3 non-hematologic toxicity as defined in CTCAE v5.0 excluding the follows:
  ∘ Fatigue, infusion reaction and alopecia,
  ∘ Nausea, vomiting, diarrhea and electrolyte imbalances responsive to appropriate medications <7 days
- Any of the Grade 4 hematologic adverse events for ≥5 days excluding febrile neutropenia
- Febrile neutropenia
- Requiring discontinuing imatinib mesylate for at least 8 days due to an adverse event
- Any severe or life-threatening complication or abnormality not defined in the CTCAE v5.0 that is attributable to the therapy

### 2.9 Treatment in phase II study

A sample size of 7 in each stage of the 2-stage design is required to detect efficacy. Eligible patients receive the dose determined in phase I cohort. The subject of the efficacy evaluation includes those who received treatment with RD in the phase I study. The cycle length of regimen is 21 days. Each patient will receive 4 cycles of imatinib mesylate and pembrolizumab combination therapy and assessed for clinical efficacy at week 12 (±7 days) at the site per RECIST 1.1. After evaluating 7 patients for response in 4 cycles of the first stage, the trial would be terminated if no patient had a CR, PR. If at least one patient met these criteria, an additional 7 patients would be enrolled for the second stage. A total of 14 (16 at maximum) patients including those who received treatment with RD in the phase I study will be assessed. At the end of the trial, if the total number of responses (CR + PR) is 3 or more, the combination would be considered promising. Each patient who shows non-PD, they are allowed to continue with combination therapy for up to 2 years. Patients will be assessed for response criteria every 4 cycles.

### 2.10 Patient discontinuation criteria

Patient must be discontinued from the trial for any of the following reasons:

- Enrollment into the trial revealed inappropriate
- Confirmed radiographic disease progression
- Meeting the protocol specified criteria for discontinuation in phase I and II study
- Unacceptable severe adverse events at the discretion of the investigator
- The patient withdraws consent for treatment due to adverse events
- The patient withdraws consent for treatment for any reason other than adverse events
- Investigator's decision to withdraw the subject

### 2.11 Statistical analysis

Statistical analyses and reporting will be conducted in accordance with the Consolidated Standards of Reporting Trials guidelines, with the primary analyses based on the intent-to-treat principle without imputing any missing observations. All efficacy analyses will be based primarily on the full dataset, which includes all patients who have received at least one dose of pembrolizumab and imatinib mesylate and were in accordance with the study protocol. Safety analysis will be conducted using data from the safety analysis population. For baseline variables, summary statistics will be performed using frequencies and proportions for categorical data and means and standard deviations for continuous variables. The primary efficacy endpoint of the study is ORR after 4 cycles from baseline, considering a response threshold of 5% (H_0_, null hypothesis) and an expected response rate of 30% (H_1_, alternative hypothesis). A minimum of 14 patients are required to achieve a 10% two-sided type I error and 80% power based on the exact binomial distribution. A phase II target sample size of 16 was set to account for an expected patient dropout of 10%. Together with maximum sample size of phase I, a total of 22 patients will be expected. All statistical analyses will be performed using SAS software version 9.4 (SAS Institute).

## 3 Discussion

This is an open-labelled single-arm clinical trial of the use of imatinib mesylate and pembrolizumab combination therapy for Japanese metastatic *KIT*-mutated melanoma patients who failed ICIs treatment. The rationale of this study relies on the several previous clinical and basic evidence showing the enhancement of the antitumor activity by KITi administration. KITi promoted immune responses by selectively reducing the immunosuppressive monocytic MDSC and by restoring CD8+ and CD4+ T-cell populations in mice.^[13]^ Furthermore, it was recently reported that anti-PD-1 therapy response in melanoma patients is modulated by the presence of tumor-infiltrating mast cells and that combining anti-PD-1 therapy with imatinib resulted in the depletion of mast cells and complete regression of tumors in humanized-mouse melanoma model.^[14]^ Clinically, one case report has shown that metastatic mucosal melanoma of the vulva harboring *KIT* mutation showed response to sequential administration of imatinib mesylate after anti-PD-1 antibody.^[15]^ In addition, another report showed that co-administration of imatinib mesylate and pembrolizumab in an anti-PD-1 monotherapy failed *KIT*-mutation positive melanoma patient achieved complete remission for almost 12 months.^[16]^ Based on these clinical and biological observations, we hypothesize that imatinib mesylate and anti-PD-1 antibody combination will show a clinical benefit in *KIT*-mutant melanoma even it has resistant to ICIs therapy.

This study has several limitations. First, the number of patients is small to assess the true safety and efficacy of imatinib mesylate and pembrolizumab combination. Second, differences in the clinical response to imatinib mesylate are expected among the study participants due to the diversity of mutational status of *KIT* gene as reported in the four phase II trials of imatinib mesylate monotherapy for melanoma; ORR of melanoma patients with *KIT* mutations in exon 11 or 13 was 24.4% while that for the patients with alterations in other gene loci was 19.4%.^[17,18]^ Third, this study uses a single arm design, and therefore does not demonstrate the superiority of imatinib mesylate and pembrolizumab combination over imatinib mesylate monotherapy. Nevertheless, if the results of this study show promising efficacy and acceptable safety profile, it will contribute to the development of novel treatment option for patients with *KIT*-mutant melanoma that are refractory to standard therapy.

## Acknowledgments

We would like to thank all members of study operation office and data center. This research was supported by Japan Agency for Medical Research and Development (AMED) under Grant Number JP21lk0201137.

## Author contributions

IH and KT contributed to the writing of the manuscript. T.F. is the principal investigator and responsible for the organization and coordination of the trial. All authors contributed to the management and administration of the trial. T.F. conceived the idea for the project and designed the study.

**Writing – original draft:** Ikuko Hirai.

**Writing – review & editing:** Keiji Tanese, Keitaro Fukuda, Takayuki Fusumae, Yoshio Nakamura, Yasunori Sato, Masayuki Amagai, Takeru Funakoshi.
